# Whole Exome Sequencing reveals new candidate genes in host genomic susceptibility to Respiratory Syncytial Virus Disease

**DOI:** 10.1038/s41598-017-15752-4

**Published:** 2017-11-21

**Authors:** Antonio Salas, Jacobo Pardo-Seco, Miriam Cebey-López, Alberto Gómez-Carballa, Pablo Obando-Pacheco, Irene Rivero-Calle, María-José Currás-Tuala, Jorge Amigo, José Gómez-Rial, Federico Martinón-Torres, GENDRES network, Antonio Justicia-Grande, Beatriz Morillo, Lorenzo Redondo-Collazo, Carmen Rodríguez-Tenreiro, Ruth Barral-Arca, Sara Pischedda, José Peña-Guitián, Carmen Curros Novo, Miriam Puente-Puig, Rosaura Leis-Trabazo, Nazareth Martinón-Torres, José María Martinón-Sánchez, Máximo Francisco Fraga-Rodríguez, José Ramón Antúnez, Enrique Bernaola-Iturbe, Laura Moreno-Galarraga, Jorge Álvarez, Teresa González-López, Delfina Suarez-Vázquez, Ángela Vázquez Vázquez, Susana Rey-García, Francisco Giménez-Sánchez, Miguel Sánchez Forte, Cristina Calvo-Rey, María Luz García-García, Ignacio Oulego-Erroz, David Naranjo Vivas, Santiago Lapeña, Paula Alonso-Quintela, Jorge Martínez-Sáenz de Jubera, Estibaliz Garrido-García, Cristina Calvo Monge, Eider Oñate-Vergara, Jesús de la Cruz Moreno, Maria del Carmen Martínez-Padilla, Manuel Baca-Cots, David Moreno-Pérez, Susana Beatriz-Reyes, María Cruz León-León

**Affiliations:** 1 Unidade de Xenética, Departamento de Anatomía Patolóxica e Ciencias Forenses, Instituto de Ciencias Forenses, Facultade de Medicina, Universidade de Santiago de Compostela(USC), Galicia, Spain; 2 GenPoB Research Group, Instituto de Investigaciones Sanitarias (IDIS), Hospital Clínico Universitario de Santiago de Compostela (SERGAS), Galicia, Spain; 3 Translational Pediatrics and Infectious Diseases, Hospital Clínico Universitario de Santiago, Santiago de Compostela, Spain; 4 GENVIP Research Group (www.genvip.org), Instituto de Investigación Sanitaria de Santiago, Galicia, Spain; 5 Área Asistencial Integrada de Pediatría, Hospital Clínico Universitario, Santiago de Compostela, Galicia, Spain; 6 Biobank, Servicio Anatomía Patológica, Hospital Clínico Universitario, Santiago de Compostela, Galicia, Spain; 7 Servicio de Pediatría, Hospital Materno Infantil Virgen del Camino, Pamplona, Navarra Spain; 8 Departamento de Pediatría, Complejo Hospitalario Universitario de Orense, Galicia, Spain; 9 Servicio de Pediatría, Hospital de la Inmaculada de Granada, Andalucía, Spain; 10 Servicio de Pediatría, Hospital Torrecárdenas, Almería, Andalucía, Spain; 11 Servicio de Pediatría, Hospital La Paz, Madrid, Spain; 12 Servicio de Pediatría, Hospital Severo Ochoa de Madrid, Madrid, Spain; 13 Servicio de Pediatría, Complejo Asistencial Universitario de León, Castilla-León, Spain; 14 Servicio de Pediatría, Hospital de Donostia, San Sebastián, País Vasco Spain; 15 Servicio de Pediatría, Complejo Hospitalario de Jaén, Andalucía, Spain; 16 Servicio de Pediatría, Hospital Quirón, Málaga, Andalucía Spain; 17 Servicio de Pediatría, Hospital Carlos Haya, Málaga, Andalucía, Spain; 18 Servicio de Pediatría, Hospital Virgen de la Arrixaca, Murcia, Spain

## Abstract

Respiratory syncytial virus (RSV) is an important cause of serious lower respiratory tract disease in infants. Several studies have shown evidence pointing to the genome of the host as an important factor determining susceptibility to respiratory disease caused by RSV. We sequenced the complete exomes of 54 patients infected by RSV that needed hospitalization due to development of severe bronchiolitis. The Iberian sample (IBS) from The 1000 Genomes Project (1000G) was used as control group; all the association results were pseudo-replicated using other 1000G-European controls and Spanish controls. The study points to SNP rs199665292 in the olfactory receptor (OR) gene *OR13C5* as the best candidate variant (*P*-value = 1.16 × 10^−12^; OR = 5.56). Genetic variants at HLA genes (*HLA-DQA1*, *HLA-DPB1*), and in the mucin 4 gene (*MUC4*) also emerge as susceptibility candidates. By collapsing rare variants in genes and weighing by pathogenicity, we obtained confirmatory signals of association in the OR gene *OR8U1*/*OR8U8*, the taste receptor *TAS2R19*, and another mucin gene (*MUC6*). Overall, we identified new predisposition variants and genes related to RSV infection. Of special interest is the association of RSV to olfactory and taste receptors; this finding is in line with recent evidence pointing to their role in viral infectious diseases.

## Introduction

Respiratory syncytial virus (RSV) was first isolated from chimpanzees in 1956^1^. Soon thereafter it was observed in young children and recognized as an important cause of serious lower respiratory tract disease in infants and young children, such as pneumonia and bronchiolitis. Today, RSV infection is the single most important cause of lower respiratory tract infections and hospital admissions during infancy and childhood. While most children are infected by the age of two years, only a minor proportion of them need hospitalization due to development of severe disease^2^. Furthermore, RSV also causes repeated infections and significant disease throughout life. Treatment is generally based on supportive care and includes oxygen therapy; after more than 60 years since its discovery, no effective vaccine is available yet^3^. There are several risk factors that predispose children to severe bronchiolitis and pneumonia, including premature birth, congenital heart disease, T-cell immunodeficiency, and chronic lung disease. However, most of the children suffering severe RSV disease are apparently healthy without known identifiable risk factors^4^. At the same time, age and sex are important independent risk factors for serious RSV disease^5^.

There is a growing body of evidence indicating association of RSV disease susceptibility with ethnicity, and with a family history of asthma^6^. Moreover, a few recent studies indicate that susceptibility to RSV infection in mice can be genetically determined^7,8^. The study by Stark *et al*.^7^ suggested that susceptibility to RSV is likely to be a multigenetic trait (as with other infectious diseases^9,10^) while some authors have suggested that host genetic variation could play a role in the severity of RSV disease. For instance, Thomsen *et al*.^11^ reported significantly higher concordance rates in the susceptibility to severe RSV infection in identical twin pairs, when compared to fraternal twin pairs.

Most genetic studies hitherto carried out in the context of RSV disease have focused on a candidate-gene based strategy. There are only a few case-control association and family-based studies performed on human cohorts reporting several candidate genes that could predispose to RSV infection^12^. Several genes seem to be related to host genetics on innate defense given that primary RSV disease occurs before the development of specific acquired immunity and in the presence of maternal antibody. The genes related to the hydrophilic surfactant proteins A1, A2 and D, namely, *SFPA1*, *SFPA2*, and *SFPD*, are considered to play a major role in the pulmonary defense mechanism and therefore in RSV infection^13^. The Toll-like receptor 4 gene (*TLR4*) regulates innate and adaptive immune response by recognizing pathogen-associate molecular patterns and could also play a crucial role in RSV infection^14^. Inoue *et al*.^15^ reported the association of *CD14* variants with the development of RSV bronchiolitis in the Japanese population. Several authors suggested the involvement of cytokine genes in the association of RSV disease. For instance, Th1 and Th2 cytokine genes such as *IL4*, *IL13*, and *IL5*, have been found to be related to RSV disease severity in a Korean cohort of hospitalized children^16^. The study by Janssen *et al*.^17^ signaled several innate immunity genes (*VDR*, *JUN*, *IFNA5* and *NOS2A*) as candidate genes in RSV infection^17^.

Genome-wide association studies (GWAs) have been very popular to disentangle the genetic component of complex multifactorial diseases; however, little effort has been devoted to genome-wide strategies for the study of the genetic susceptibility to RSV infection so far. Very recently, Pasanen *et al*.^18^ carried out the first genome-wide analysis of bronchiolitis patients; the study indicated suggestive association signals for several SNPs, including rs269094 located in gene *KCND3*, which has previously been linked to asthma^19^. GWAs studies usually target common and noncoding genetic variants (MAF ≥ 5%) whose effects are small (relative risk~1.5), and therefore cannot cover rarer genetic factors that could be associated to RSV disease. It is therefore possible that SNPs usually genotyped in GWAs capture only a small proportion of disease heritability, while an important part of this heritability could reside in rare variants. In contrast, next generation sequencing (NGS) technologies applied to the study of exomes (whole exome sequencing; WES) allow exploring the majority of non-synonymous coding genetic variants in cohorts of patients. Sequencing the complete exome of patients enables the identification of all genetic variation existing in a gene (most of human variation consists of rare variants), and therefore there is no need to rely on linkage disequilibrium (LD) to tag uncovered causal SNPs. In addition, studies based on the selection of extreme phenotypes for the identification of relevant clinical variants have several advantages when compared to those studies that do not take into account clinical sub-phenotypes^20^. Thus, a modest sample size can achieve the necessary statistical power to identify new candidate genes, because the frequency of alleles that contribute to disease would be enriched in one or both phenotypic extremes and their genetic effect could be higher^21^.

At the same time, owing to the characteristics of the patients suffering from pediatric infectious diseases, reaching sufficiently large sample sizes to carry out powerful studies is typically a challenging task. Therefore, sequencing exomes of selected extreme phenotypes emerged as the most promising strategy for the analysis of genetic risk factors underlying pediatric infectious diseases, allowing focusing on protein-altering variants. This variation is enriched for causal effects^22^.

The present study aims to explore genome-wide variation of severe phenotypes of RSV disease patients compared to healthy controls using a WES strategy of selected phenotypes. WES allows exploring common and rare variation involved in host susceptibility to RSV infection.

## Material and Methods

### Study design and inclusion criteria

Patients were selected from a cohort prospectively recruited under an observational study run in Spain through a national hospital-based research network for pediatric respiratory research: GENDRES (Genetics, Vitamin D and Respiratory Infections Research Network – www.gendres.org). This network includes 13 Spanish tertiary hospitals. Eligible study participants in GENDRES were previously healthy children under 14 years of age admitted to a participating hospital with an acute respiratory infection diagnosis. From Dec 2009 to Jun 2014, a total of 551 subjects were recruited by this network. For the present study, hospitalized children from 0 to 3 years of age with confirmed diagnosis of RSV bronchiolitis were selected, provided that (i) written informed consent was available from a parent or legal guardian, (ii) at least a DNA sample was collected, and (iii) the minimum mandatory demographic and clinical data set was available.

RSV status was determined in all patients by routine diagnostic investigations as part of clinical care in the referring hospital. Only patients with immunofluorescence test-confirmed RSV infection and no features of co-existing bacterial infection at admission according to clinical data, inflammatory markers, radiological findings and/or appropriate cultures were included in this study. Patients included were diagnosed as acute bronchiolitis due to RSV according to the ICD-10 definition.

A blood sample was collected from each patient for DNA extraction and posterior sequencing analysis. DNA of these patients was extracted using the Wizard® Genomic DNA Purification Kit (Promega).

For the present study, 59 children with confirmed diagnosis of RSV were finally selected from the GENDRES cohort, although only 54 were used after applying different methodological filters. The patients’ main characteristics are summarized in Table 1.

**Table 1:** Summary of the demographic and clinical characteristics of the study cohort.

Variables	RSV (*n* = 54)
Demographic characteristics	
Sex (male)^a^	61.1% (33/54)
Age (years)^b^	0.2 (0.1–0.7)
Past family history	
Asthma^a^	29.6% (16/54)
Respiratory problems^a^	33.3% (18/54)
Medical history	
Pneumococcal vaccination status^a^	
PVL 7	5.7% (3/53)
PVL 10	1.9% (1/53)
PVL 13	41.5% (22/53)
Clinical data	
Hospital length of stay (days)^b^	7 (5–10)
PICU admission^a^	50% (19/38)
PICU (days)^b^	6 (4–8.25)
Respiratory distress^a^	
Severe	27.8% (15/54)
Moderate	57.4% (31/54)
Mild	14.8% (8/54)
Respiratory support^a^	33.3% (18/54)
Mechanical	3.7% (2/54)
Non-invasive	29.6% (16/54)
ReSVinet score^b^	11 (8–13.5)
Wood Downes score^b^	6 (5–7)
Oxygen^a^	79.6% (43/54)
Bacterial super-infection^a^	13.2% (7/53)

^a^Percentage and number of patients.

^b^Median (interquartile range).

The study was approved by the Ethical Committee of Clinical Investigation of Galicia (CEIC ref. 2010/015). The study was conducted according to the principles of the Declaration of Helsinki and in accordance with all applicable Spanish normative, namely, Law for Biomedical Research (Law 14/2007-3 of July), Law 41/2002 of Autonomy of the Patient, Decree SAS/3470/2009 for Observational Studies and Law 15/1999 of Data Protection.

Control groups for genomic comparisons were selected from The 1000 Genomes Project (http://www.1000genomes.org; hereafter 1000G); see details below. The 1000G European population sets were used for association tests: the Iberian control group (IBS) was used as the main control group; while CEU, GBR and TSI were used as additional control groups (see more details below).

### Exome sequencing

Samples were prepared according to Agilent’s SureSelect Protocol Version 1.2. Enrichment was carried out according to Agilent SureSelect protocols. Concentration of each library was determined using Agilent’s QPCR NGS Library Quantification Kit (G4880A). Samples were pooled prior to sequencing with each sample at a final concentration of 10nM.

Sequencing was performed on the Illumina HiSeq2000 platform using TruSeq v3 chemistry. Mapping and alignment were carried out as follows. First, read files (Fastq) were generated from the sequencing platform using the manufacturer’s proprietary software. Reads were mapped to their location in the reference human genome (hg19/b37) using the Burrows-Wheeler Aligner (BWA) package, version 0.6.2. Local realignment of the mapped reads around potential insertion/deletion (indel) sites was carried out with the Genome Analysis Tool Kit (GATK) version 1.6. This algorithm ensures that the alignment has the minimum number of mismatching bases across the reads; the main effect of this step is to reduce false-positive single nucleotide variant calls around indels, and hence determine indel length more accurately. Duplicate reads were marked using Picard version 1.104. This removes reads likely to be the result of PCR bias. Such PCR artifacts can introduce false-positive SNP calls. Reads were not removed from the alignment but they were not considered further in the analysis.

Additional BAM file manipulations were performed with Samtools 0.1.18. Base quality (Phred scale) scores were recalibrated using GATK’s covariance recalibration. This improves the accuracy of the base quality metrics, which in turn enhances the quality of variant calls.

Exome sequencing was carried out in Oxford Gene Technology (OGT; http://www.ogt.co.uk) using Agilent’s SureSelectXT Human All Exon V5 exome design without UTRs. The raw data were processed entirely at the laboratory of Santiago de Compostela (Spain). The average on-target coverage found on all samples was 64.23×.

### Quality control

One additional sample was processed following exactly the same steps as the rest of the samples, including WES. The two exome sequences were compared and the coincidence of the sequencing results was 99.999%.

In addition, we carried out an independent genotyping of the SNP variants that turned to be statistically associated in our case-control associated study between RSV and IBS controls, including both SNPs obtained from individual association tests and those SNPs in genes statistically significant under a burden association test. We therefore designed three SNP multiplex reactions for genotyping using the MassARRAY SNP genotyping system (Sequenom, San Diego, CA, USA) and using the iPLEX GOLD (Agena Bioscience) assay to increase plexing efficiency and flexibility. Since this genotyping effort aimed at independently evaluating the quality of exome sequencing results, only those SNPs meeting the highest standards required by the Assay Design 3.1 software were incorporated to the final plexes and successfully genotyped (*n* = 31).

The concordance between MALDI-TOFF assays genotyping and parallel sequencing was 98.0% for all the genotypes (and 98.1% for those carriers of the minor alleles at the individual associated variants found in the present study). The very low estimated sequencing error rate in our exomes (0.001%) coupled with the sequencing error rate of the Mass Array platform (2.3%;^23^), add solid support to the variation detected in the present study.

### Variant detection, annotation and *in silico* assessment of their pathogenicity

Variants were detected using GATK v3.4 best practice for multi-sample calling, which increases the detection and genotyping power of this already very sensitive variant caller. Genomic VCF files were obtained for each sample using the HaplotypeCaller algorithm, and joint genotyping was performed using the GenotypeGVCFs algorithm. Variant quality scores were then recalculated using the VQSR algorithm to allow the filtering of outlying variants by improving the sensitivity *vs*. specificity balance. Variants were annotated using ANNOVAR^24^, and using gene and gene function data from Ensembl (http://www.ensembl.org/index.html). This procedure makes it possible to determine which genes and transcripts are affected by the variations, and whether these variants are likely to cause significant functional problems. Known variants from dbSNP (Release 135) were annotated within the dataset so that novel variants with serious predicted consequences may be rapidly identified.

Different scoring systems were applied to the annotated variants in order to measure the pathogenicity/deleteriousness of single nucleotide variants as well as insertion and deletion variants in the exomes (PolyPhen^25^, SIFT^26^, GERP^27^, etc); Table S1. Besides those methods that exploit a single information type (such as conservation) and/or are restricted in scope (e.g. to missense changes) we also obtained the Combined Annotation Dependent Depletion (CADD), a score that integrates multiple annotations into one metric by contrasting variants that survived natural selection with simulated mutations^28^. CADD can quantitatively prioritize functional, deleterious, and disease-causal variants across a wide range of functional categories, effect sizes and genetic architectures and can be used to prioritize causal variation in both research and clinical settings. The linear kernel support vector machine-based algorithm used in CADD analysis has been improved by using a deep neural network which also considers nonlinear effects; this algorithm is known as Deleterious Annotation of Genetic Variants (DANN)^29^, and it also provides a score.

### Population-based and statistical analysis

Identity-by-state values were computed from SNP data using PLINK^30^. In order to allocate the genome variability observed in our cohort of patients, we carried out Multidimensional Scaling (MDS) analysis on a matrix of pairwise individual identity-by-state values, and in the context of main continental variation. MDS was performed using the function *cmdscale* (library stats) from R (http://www.r-project.org). Next, we additionally investigated admixture patterns in our patients using different source populations representing main continental regions (sub-Saharan Africa, East Asia, and Europe). Population data for comparison was retrieved from 1000G with the assistance of previous bioinformatics developments^31,32^. First, all variant files were downloaded from the 1000G public ftp (ftp://ftp.1000genomes.ebi.ac.uk/vol1/ftp/release/20110521/); next, we followed the procedures described previously for the processing of these data^33^.

Maximum likelihood estimation of individual ancestries from multi-locus SNP data was carried out using ADMIXTURE^34^.

We also explored the genome of all patients and controls for potential familial relationship as done previously^35,36^.

Single-point association analyses were carried out using the Fisher exact test. The 1000G European datasets for Iberia (IBS), Great Britain (GBR), Tuscany (TSI), and CEU (USA-European ancestry, CEU) were used as controls. The possible confounding effect of population stratification was estimated by calculating the genomic inflation factor (lambda or λ) from the data^37^; λ is the ratio of the median of the empirically observed distribution of the chi-squared values to the expected median. As such, it provides a way to quantify the extent of the genome inflation and the excess false-positive rate. This uniform correction can over-adjust or under-adjust certain SNPs depending on the ancestral information of individual variants; however, as shown below, we first demonstrate that the samples analyzed, cases and controls, are genetically homogeneous and share a common European ancestral background.

In addition, SNP association analyses were carried out, collapsing variants by genes and taking into account several parameters that could indicate potential pathogenicity (weighted burden tests). The procedure was carried out as follows. For an individual *j* and a Region of Interest (ROI) with *K* biallelic positions (being K > 5), we define *x′*_*ij*_ as the number of variants at position *i* for subject *j*. This parameter can take values 0, 1 or 2. Our approach, modified from^38^, follows the following steps:

- For each subject, we compute ${x}_{j}^{^{\prime} }=\frac{1}{K}{\sum }_{i=1}^{K}dan{n}_{i}\,{x}_{ij}^{^{\prime} }$.
- The sum of ranks of ${x}_{j}^{^{\prime} }$ is computed for affected subjects $r=\frac{1}{K}{\sum }_{j\in affected}\,{x}_{ij}^{^{\prime} }$. To deal with the possible ties, we assign the average rank position for the tied individuals.
- The affection status is permuted B times, and r*_b_ values are computed to simulate the distribution under the null hypothesis.
- The *P*-value is calculated considering the normal distribution with the permutated mean $(\widehat{{\mu }_{B}}=\frac{1}{B}{\sum }_{i=1}^{B}{r}_{i}^{\ast })$ and permutated standard deviation $(\widehat{{\sigma }_{b}}=\sqrt{\frac{1}{B}{\sum }_{i=1}^{B}{({r}_{i}^{\ast }-\widehat{{\mu }_{B}})}^{2}})$.

Collapsing variant analysis was carried out on all variants observed in exomes, and separately on common, rare, and non-synonymous variants.

A Bonferroni correction was used in order to account for multiple hypotheses. Most of the association test analyses were carried out using in-house R and Perl (http://www.perl.org) scripts.

Haploview was used to display LD patterns between SNPs within genes^39^. Finally, Gene Ontology (http://www.geneontology.org) analysis of statistically significant genes was carried out on PANTHER^40^.

## Results

### Clinical features of patients

A total of 54 patients of European ancestry were sequenced in the present study. The cohort characteristics are summarized in Table 1. From this group, 61.1% of the subjects were male with a median age of 0.2 years (0.1–0.7). Family history showed respiratory problems in 18 (33.3%) of the subjects, 16 (29.6%) of them being asthma. Prematurity was part of the medical history of two of the subjects. The main reason for hospital admission was respiratory distress (*n* = 53). We classified subjects clinically with regards to the level of respiratory affection: 8 (14.8%) were mild, 31 (57.4%) were moderate, and 15 (27.8%) were severe. Wood-Downes and ReSVinet scores were also applied to assess patient severity^41^. The Wood-Downes score divided the patients in 11 (20.4%) clinically mild, 30 (55.6%) moderate and 13 (24.1%) severe subjects, with an average score of 6 (5–7). The ReSVinet score distinguished between severe (≥13) and moderate-mild, with an average score of 11 (8–13.5). During admission, supplementation with oxygen was needed in 43 (79.6%) cases. Corticoids (systemic or inhaled) were used in eight (14.8%) subjects. 19 (35.2%) of the subjects were admitted to PICU, with a median stay in days of 6 (4–8.3). Ventilatory support was applied in 18 (33.3%) patients, 16 (29.6%) were non-invasive and two (3.7%) invasive ventilation. Secondary to hemodynamic instability inotropic drugs were deemed necessary in 21 (38.9%) subjects. The median length of hospital stay was 7 days (5–10). No major sequelae or mortality was present at discharge.

### Ancestry analysis of RSV patients

**Figure 1:**
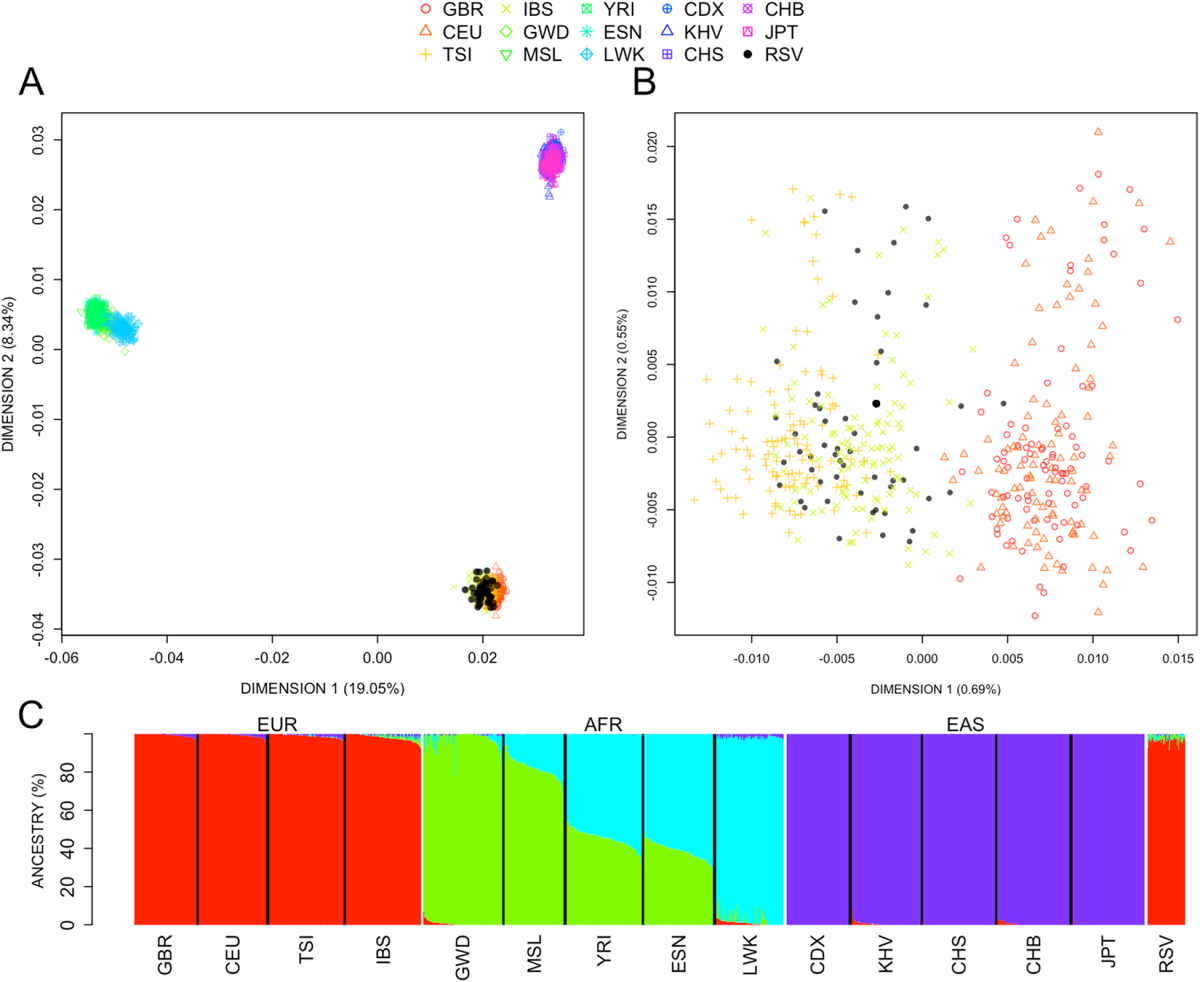
(**A**) MDS analysis carried out on a matrix of pair-wise individual identity-by-state values between RSV patients *vs*. different 1000G population reference sets (see legend inset). (**B**) Enlarged detail of the European cluster observed in (**A**). (**C**) Admixture analysis of the samples analyzed in (**A**).

Analysis of the population-genetic characteristics of patients is important in order to detect possible genomic outliers. We first carried out a MDS to a continental scale using 1000G populations as references. As shown in Fig. 1A and B, our cohort of patients shows patterns of variation that fit perfectly in the European cluster in both the first dimension (accounting for ~19% of the variation) and the second dimension (~8%) of the MDS, clustering together with other samples of European ancestry. A good fit exists between RSV cases and IBS controls when other dimensions of the MDS are examined (Figure S1). In addition, we carried out an admixture analysis in order to corroborate the patterns observed in the MDS. As expected, ancestry of our cohort of patients is virtually 100% European (Fig. 1C).

We finally investigated potential relationships in our cohort of patients in order to avoid potential artifacts and interferences with the association analysis. The data show that there are no close relationships among them.

### Annotated variant and SNP association analyses

A total of 108,800 variants (passing the filters indicated above) were annotated from the exome data, of which 106,711 remained after filtering by Hardy-Weinberg equilibrium (*P*-value > 0.005 in all cases, control groups individually, and control groups merged in a single group). 81,461 of the filtered variants salasare exonic, of which 41,596 are non-synonymous (Table 2). All these variants have an average genotyping rate >99.99%.

**Table 2:** Description of variants found in our patients.

Variation	*N*
Downstream	6
Exonic	81461
Exonic/splicing	54
Intergenic	38
Intronic	365
ncRNA_exonic	5845
ncRNA_exonic; splicing	5
ncRNA_intronic	497
ncRNA_splicing	3
Splicing	52
Upstream	18
Upstream; downstream	3
UTR3	10958
UTR5	7390
UTR5/UTR3	16
Non-synonymous SNP	41596
Stopgain	416
Stoploss	43
Synonymous SNP	38401
Unknown	1059

Although MDS and admixture analysis do not show evidence of population stratification between cases and controls, we computed the inflation factor on *P*-values existing between cases and the different European controls used for the association analysis. Given that λ was below 1, it was not necessary to control association tests for inflation.

**Figure 2:**
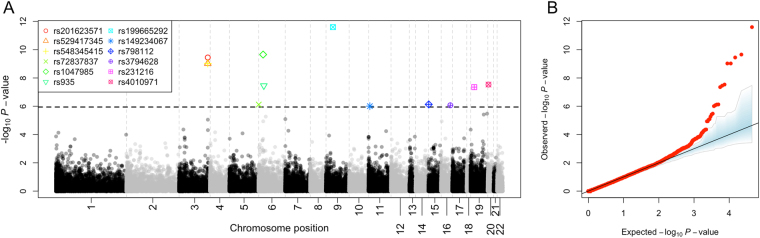
(**A**) Manhattan plot of common variants observed in RSV patients. (**B**) QQ-plot of *P*-values for common variation observed in IBS controls. *P*-values obtained under a permutation approach (1,000 permutations) are shown in blue.

**Figure 3:**
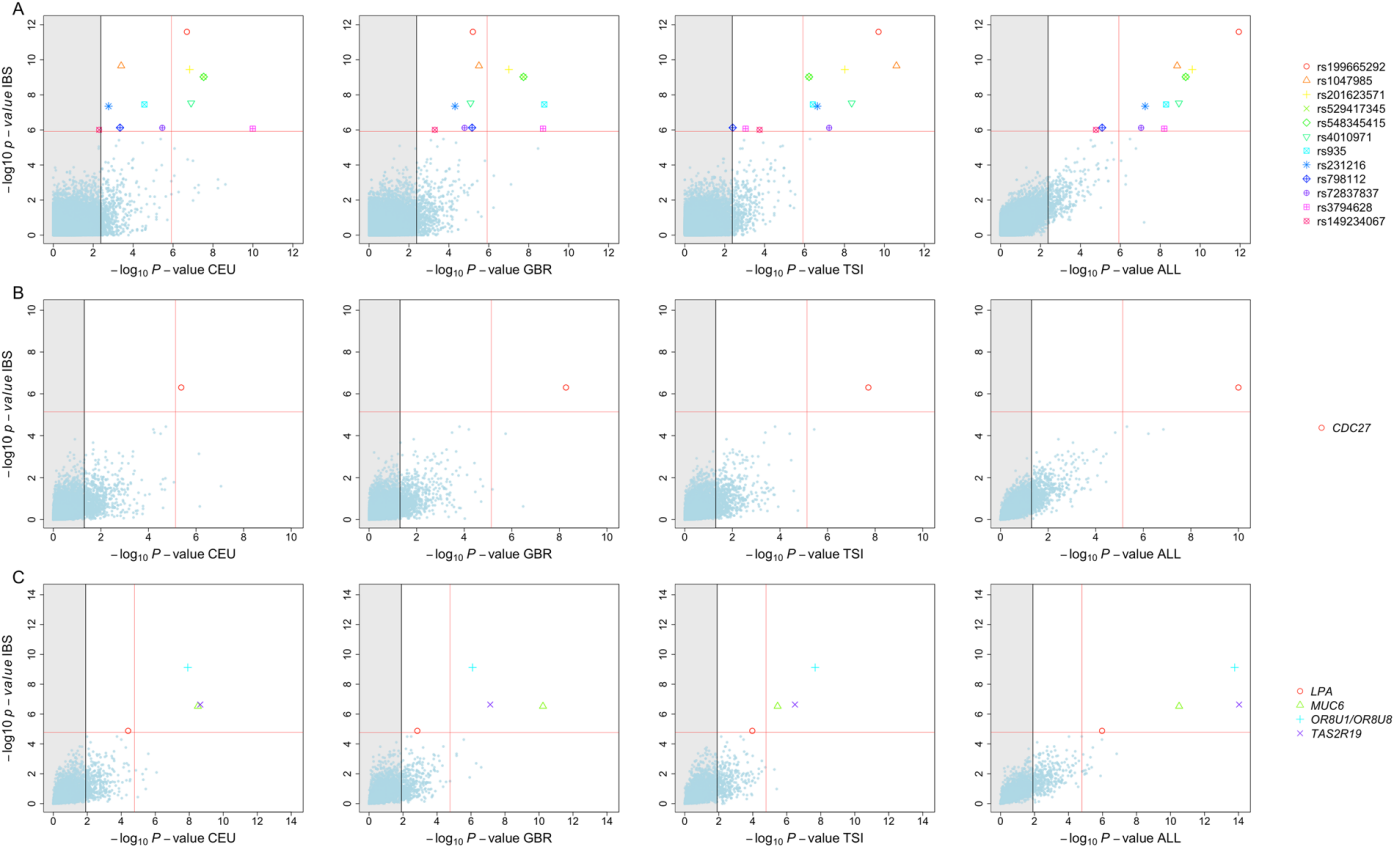
(**A**) *P*-values of SNP association between RSV patients and controls in different control groups and in the merged control group “ALL” (IBS + CEU + GBR + TSI). (**B**) *P*-values of gene burden association test between RSV patients and controls in different control groups and in the merged control group “ALL” considering all variants in genes. (**C**) *P*-values of gene burden association test between RSV patients and controls in different control groups and in the merged control group “ALL” considering only rare variants in genes. The vertical grey shadow in the figures indicates the threshold for the adjusted Bonferroni *P*-value according to the number of independent tests considering CEU, GBR, TSI, and ALL as additional control groups of the best SNPs (*n* = 12) and gene candidates (one gene when collapsing common variants and four genes when collapsing rare variants). The red vertical line indicates the genome Bonferroni threshold considering genes and all the common SNPs that are shared between cohorts.

Single association tests were carried out for common variants with minor allele frequencies (MAF) ≥ 0.05 (Table 3; Table S1). The QQ-plot in Fig. 2B shows a few SNPs with very low *P*-values that differ from the expected distribution of *P*-values under a uniform distribution. To further test for the robustness of the statistical significance of these associated SNPs, a simulation approach was conceived that computes the *P*-values for each SNP marker after permuting the case-control status of all samples. Under 1,000 permutations, we observe a very good fit between the theoretical expected *P*-values and the permuted ones (Fig. 2B). A total of 12 SNPs have *P*-values that are below the conservative Bonferroni adjustment (Fig. 2A) in IBS. The signal of association was explored in the other three control groups (CEU, GBR, and TSI). All the markers surpassed the multiple test threshold (Bonferroni adjustment considering 12 variants) in all control groups. Moreover, four of them (rs199665292, rs201623571, rs529417345, and rs548345415; Table 3) yielded statistical *P*-values below the genome Bonferroni threshold (Fig. 3; see also Figure S2); only two of them (rs798112, and rs3794628) were below the genome-wide Bonferroni adjustment when merging all the control groups (including IBS) in a single one (hereafter “ALL”); Fig. 3A. To test for the suitability of CEU, GBR, and TSI as control groups of our RSV cohort, we carried out an association test between SNP variations in IBS *vs*. these other three control groups. Only 18, 10, and 7 SNPs out of 108,800 variants were statistically significant under a Bonferroni adjustment, and none of these few SNPs coincided with the 12 SNPs observed to be statistically significant between RSV and the control groups (Figure S3). This shows that the four cohorts are not different from the point of view of an association test.

**Table 3:** Association test of common variants.

SNP	Chr	Position	Gene	Gene region	Exonic function	OR_IBS_	*P*-value_IBS_	OR_ALL_	*P*-value_ALL_	P-value_EC_
rs199665292	9	107361452	*OR13C5*	exonic	Synonymous	9.09	2.53 × 10^−12^	5.56	1.16 × 10^−12^	3.53 × 10^−15^
rs1047985	6	32605216	*HLA-DQA1*	UTR5	—	0.05	2.21 × 10^−10^	0.06	1.43 × 10^−09^	2.50 × 10^−06^
rs201623571	3	195508393	*MUC4*	exonic	Non-synonymous	0.08	3.55 × 10^−10^	0.10	2.48 × 10^−10^	—
rs529417345	3	195508416	*MUC4*	exonic	Synonymous	0.03	9.40 × 10^−10^	0.03	5.31 × 10^−10^	—
rs548345415	3	195508418	*MUC4*	exonic	Non-synonymous	0.03	9.40 × 10^−10^	0.03	5.31 × 10^−10^	—
rs4010971	20	29637822	*MLLT10P1*	ncRNA_exonic	—	0.19	2.92 × 10^−08^	0.20	1.16 × 10^−09^	3.77 × 10^−06^
rs935	6	33054579	*HLA-DPB1*	UTR3	—	0.14	3.51 × 10^−08^	0.15	5.09 × 10^−09^	—
rs231216	19	36259494	*PROSER3*	UTR3	—	5.35	4.43 × 10^−08^	3.72	5.70 × 10^−08^	9.27 × 10^−10^
rs798112	15	31110279	*HERC2P10*	ncRNA_exonic	—	0.10	7.43 × 10^−07^	0.13	8.15 × 10^−06^	4.30 × 10^−04^
rs72837837	6	15520916	*JARID2*	UTR3	—	—	7.59 × 10^−07^	—	9.09 × 10^−08^	—
rs3794628	16	88729788	*SNAI3-AS1*	ncRNA_exonic	—	0.14	8.52 × 10^−07^	0.13	6.29 × 10^−09^	—
rs149234067	11	33722084	*C11orf91*	exonic	Non-synonymous	0.23	9.81 × 10^−07^	0.30	1.67 × 10^−05^	—

The SNPs showing the lowest *P*-value against the IBS control group were further tested using CEU, GRB, and TSI as control groups and merging the three sample sets into a single one (“ALL” = IBS + CEU + GBR + TSI). EC refers to the exome sequencing data in the Spanish control group (*n* = 267) of Dopazo *et al*.^42^. The SNP rs199665292 has merged into rs75081605 (https://www.ncbi.nlm.nih.gov); a hyphen in column “*P*-value_EC_” indicates that there is not genetic information available in the Spanish control groups concerning this variant.

Chr = chromosome; *P*-values: Fisher’s Exact Test; OR = odds ratio.

We furthermore carried out an association test between RSV patients and the exome data of the Spanish control group (EC) in Dopazo *et al*.^42^. Only five out of the 12 candidate SNPs are present in this dataset. The five SNPs received *P*-values below the Bonferroni threshold (Table 3).

The lead SNP was rs199665292 (*P*-value_IBS_ = 2.53 × 10^−12^, OR_IBS_ = 9.09; *P*-value_ALL_ = 1.16 × 10^−12^, OR_ALL_ = 5.56), an exonic synonymous SNP located in gene *OR13C5* (Table 2). This SNP appears in ExAC browser as tetra-allelic, although two alleles appear only twice in more than >103,400 alleles; however, we only detected the two common main alleles in our cohort of patients and in all reference populations (including 1000G). The second most significant SNP, rs1047985 (*P*-value_IBS_ = 2.21 × 10^−10^, OR_IBS_ = 0.05; *P*-value_ALL_ = 1.43 × 10^−09^, OR_ALL_ = 0.06), falls within the *HLA-DQA1* gene and it is located in an UTR5 region. The next most significant variants are three SNPs, all within gene *MUC4*: rs201623571 (*P*-value_IBS_ = 3.5 × 10^−10^, OR_IBS_ = 0.08; *P*-value_ALL_ = 2.48 × 10^−10^; OR_ALL_ = 0.10), rs529417345 and rs548345415 (*P*-value_IBS_ = 9.40 × 10^−10^, OR_IBS_ = 0.03; *P*-value_ALL_ = 5.31 × 10^−10^; OR_ALL_ = 0.03); interestingly, rs201623571 and rs548345415 are non-synonymous (Table 3). Another SNP out of the 12 statistically significant ones, rs149234067 in gene *C11orf91*, is also non-synonymous.

### Weighted burden test by genes

By collapsing all variants observed in genes, a clear signal of association was observed for *CDC27*, with statistical significance bellow the genome-wide Bonferroni threshold for all European control groups individually, as well as for the merged controls (*P*-value_IBS_ = 4.97 × 10^−7^, *P*-value_ALL_ = 1.01 × 10^−10^); Table 4, and Fig. 3B.

**Table 4:** Association test of SNPs collapsed by genes and using average CADD per gene as a covariant for the association test.

Gene	Chr.	n° SNP	*P*-value_IBS_	*P*-value_CEU_	*P*-value_GBR_	*P*-value_TSI_	*P*-value_ALL_	*P*-value_EC_
*CDC27*	17	6	4.97 × 10^−07^	4.20 × 10^−06^	5.29 × 10^−09^	1.92 × 10^−08^	1.01 × 10^−10^	4.3 × 10^−11^
*OR8U1/ OR8U8*	3	9	7.60 × 10^−10^	1.22 × 10^−08^	8.10 × 10^−07^	2.14 × 10^−08^	1.69 × 10^−14^	—
*TAS2R19*	4	6	2.30 × 10^−07^	2.25 × 10^−09^	7.41 × 10^−08^	3.30 × 10^−07^	9.21 × 10^−15^	—
*MUC6*	3	18	3.00 × 10^−07^	3.10 × 10^−09^	5.85 × 10^−11^	3.46 × 10^−06^	3.12 × 10^−11^	7.84 × 10^−02^
*LPA*	19	12	1.34 × 10^−05^	3.96 × 10^−05^	1.44 × 10^−03^	1.07 × 10^−04^	1.07 × 10^−06^	1.07 × 10^−03^

*CDC27* appeared as statistically significant when analyzing common variants (MAF ≥ 0.05), while the other genes appeared as statically significant when analyzing rare variants (MAF < 0.05). The genes showing the lowest *P*-value against the IBS control group were further tested using CEU, GRB, and TSI as control groups and merging the three sample sets into a single one (“ALL” = IBS + CEU + GBR + TSI); a hyphen in column “*P*-value_EC_” indicates that there is not enough genetic information available in the Spanish control groups.

Many deleterious and disease-related variants exist at low frequency in the human genome^43^. We partitioned the variants observed in our patients into separate classes according to their MAF: rare (<0.05) and common variants (≥0.05). By collapsing common variants in genes, we did not identify any candidate gene. However, burden tests on rare variants yielded significant signatures for five genes in IBS (Table 4; Fig. 3C). The signal of association was investigated in three other control groups (CEU, GBR, and TSI). All the genes surpassed Bonferroni correction (considering four independent genes). Moreover, all these genes yielded statistical *P*-values below the genome Bonferroni threshold in the European control category ALL, and most of them (with the exception of *LPA* gene) in the European control group individually (Fig. 3). The top association signal was found for the olfactory receptor *OR8U1*/*OR8U8* (*P*-value_IBS_ = 7.60 × 10^−10^, *P*-value_ALL_ = 1.69 × 10^−14^). The other genes were, in order of significance: *TAS2R19* (*P*-value_IBS_ = 2.30 × 10^−7^, *P*-value_ALL_ = 9.21 × 10^−15^), *MUC6* (*P*-value_IBS_ = 3.00 × 10^−7^, *P*-value_ALL_ = 3.12 × 10^−11^), and *LPA* (*P*-value_IBS_ = 1.34 × 10^−5^, *P*-value_ALL_ = 1.07 × 10^−6^).

In addition, we carried out an association test between RSV patients and the exome data of the Spanish control group in Dopazo *et al*.^42^. The analysis could only be carried out on three out of the five genes found to be associated in the discovery cohort (namely those genes with a minimum number of variants per gene according to the procedures described in Material and Methods). The three genes received *P*-values below the Bonferroni threshold (Table 3), and the *CDC27* was highly significant (*P*-value_EC_ = 4.3 × 10^−11^), surpassing the genome-wide Bonferroni threshold.

For all statistically significant genes observed in these analyses, the accumulated pathogenicity was increased in cases with respect to controls.

Non-statistically significant results were obtained when computing the weighted burden test only on non-synonymous variants.

### Gene ontology: enrichment analysis of genes associated to RSV infection

To better understand the molecular function of the best candidate genes observed in the association analysis, we examined their Gene Ontogeny (GO) classification^44^. All genes signaled by the single base association analysis together with those captured by collapsing variants, were used to infer whether any biological process, cellular component, or molecular function is over- or under-represented with respect to expectations.

The biological process “antigen processing and presentation of peptide or polysaccharide antigen *via* MHC class II” was the most statistically significant under Bonferroni correction (*P*-value = 5.99 × 10^−4^), with a Fold enrichment > 100. When analyzing GO by cellular component, the category “MHC protein complex” was also found to be overrepresented (*P*-value = 1.37 × 10^−5^), again with a Fold enrichment > 100. By analyzing by protein class, the “MHC antigen” class was also significant (*P*-value = 7.58 × 10^−4^; Fold enrichment: 97.7). Finally, when analyzing the “GO biological process complete”, the class “immune response-activating cell surface receptor signaling pathway” was also found to be enriched (*P*-value = 2.09 × 10^–2^; Fold enrichment: 20.9).

## Discussion

The present study adds convincing support to our hypothesis that the genome of the hosts carries variation predisposing to severe infection by RSV. Among the new predisposition variants and genes identified, olfactory and taste receptors associations are of special interest.

By analyzing the exome of RSV infected patients we aimed to capture functional variation that could explain the association between RSV infection and the host. In order to prevent the confounding effect of population stratification, we initially carried out several population-based analyses; these analyses indicated a good match between cases and controls in terms of their genomic ancestry.

A total of 12 SNPs show genome-wide statistical significance. The top SNP falls within *OR13C5* gene, known as OR family 13 gene. This is a risk variant and was consistently significant in all control groups, including the merged European controls. Although this is a synonymous SNP, its main interest derives from the fact that another OR gene (*OR8U1*/*OR8U8*), which is a member of the OR gene family 8 and subfamily U, was found to be statistically significant when collapsing rare variants and controlling for pathogenicity. Olfactory receptors interact with odorant molecules in the nose, to initiate a neuronal response that triggers the perception of a smell. Olfactory receptors share a 7-transmembrane domain structure with many neurotransmitter and hormone receptors and are responsible for the recognition and G protein-mediated transduction of odorant signals^45^. At the same time, the nasal cavity is the natural entrance and replication of RSV host invasion. Several studies have investigated the olfactory nerve, which connects the nasal cavity directly with the central nervous system (CNS), in regard to many virus infections, including influenza A virus, herpesviruses, adenoviruses, etc. Thus, several observations have shown that neurons in the olfactory epithelium can be targeted by influenza A virus in mice^46^, ferrets^46^, and humans^47^, and that highly neurovirulent H5N1 avian influenza strains in ferrets can spread through olfactory nerves to the olfactory bulb after intranasal infection^48^. In humans, influenza virus antigens in neurons and glia in the olfactory bulbs and tracts have recently been found in an immune-compromised child^49^. RSV is not clinically feared as a neurovirulent virus, with neurological symptoms present in less than 2% of patients admitted to hospital^50^. However, Riel *et al*.^51^ suggested the interest of the nerve route not only for neurotropic viruses, but also as a shortcut for common respiratory infections, including RSV. Several viral and host factors contribute to the pathogenesis of the disease, and both acquired and genetic host factors will probably affect the pathogenesis and outcome of RSV infections, similarly to severe influenza infection in mouse models^52^. Herpes simplex virus 1 has been shown in mice model to efficiently use the olfactory neuroepithelium to infect, spread and reemerge peripherally in the skin without significant neurological disease associated^53^. Our findings of human genetic variations related to RSV infection suggest either a subclinical involvement of olfactory pathways during RSV infection, or at least a new specific pathway involved in the pathogenesis of the disease that deserves further exploration. According to Riel *et al*.^51^, although RSV and other non-neurotropic viruses do not cause severe CNS complications, they could cause subclinical or mild disease.

Kalbe *et al*.^54^ suggested that OR modulate physiological processes in human airways smooth muscle cells, which significantly contributes to the progression of chronic inflammatory airway disease, including asthma and chronic obstructive pulmonary disease (COPD); see also^55^. There is also emerging evidence suggesting that OR can express in non-olfactory tissues; for instance, Li *et al*.^56^ indicated that OR genes express in pulmonary macrophages in mice, and these genes may regulate macrophage function by regulating MCP-1 production and cell migration. According to these authors, stimulation of pulmonary macrophages with the host defense cytokine IFN-γ up regulates the expression of ORs in this murine model.

Two of the top SNPs, rs1047985 and rs935, fall within *HLA-DQA1* and *HLA-DPB1* genes, respectively, and have a protective effect. There are many studies indicating a role for HLA genes in infectious diseases in general^57,58^, and specifically with bronchiolitis^18^, as well as vaccine response^59^. The study of the transcriptome in RSV bronchiolitis has also shown a role for HLA genes^60–62^.

Three out of the 12 lead SNPs (Table 3) fall within the same gene, *MUC4*; these three variants do not constitute a strong block of linkage disequilibrium (Supplementary Data Figure S5). Another mucin gene, *MUC6*, appears as statistically significant when considering accumulated pathogenicity at rare variants. There are about 20 human mucin genes. These genes encode mucin monomers that are synthesized as rod-shape apomucin cores that are post-translationally modified by exceptionally abundant glycosylation. The dense sugar coating of mucins is very resistant to proteolysis, which may be important in maintaining mucosal barriers. There is an increased mucin production in many cancers, but mucins are also overexpressed in lung diseases such as asthma, bronchitis, COPD or cystic fibrosis. Mucins constitute an important component of the inflammatory and innate immune response although the expression of these molecules by respiratory viral infections is still largely unknown^63^. Polymorphisms in mucin genes have been identified in susceptibility to *H*. *pylori*-induced pathology in human populations^64^. A recent study of mucin expression in a human epithelial carcinoma cell line (A549) showed a strong induction of *MUC8*, *MUC15*, *MUC20*, *MUC21*, and *MUC22* mediated by RSV infection^65^. Furthermore, RSV fusion protein interacts with epidermal growth factor receptor (EGFR) in a strain-specific manner, suggesting that EGFR is a co-factor for infection and that EGFR plays a role in RSV-induced mucin expression^66^. In general, there is abundant literature relating RSV infection to upregulation of mucin genes^67–70^. Finally, another gene that could be interesting for future investigations is *TAS2R19*, it is involved in the perception of salty and bitter tastes. A recent review by Lee *et al*.^71^ pointed to an important role for taste receptors in airway innate immunity as sentinel detection systems. Also, Malki *et al*.^72^ have very recently shown that taste and olfactory receptors show differential mRNA expression in five types of blood leukocytes. Polymorphisms in another taste gene receptor (*TAS2R38*) were related to nitric oxide production and responsiveness to gram-negative bacteria^73^.

As a whole, the candidate genes signaled by the present study seem to play a role in the immune response. In this sense, the major histocompatibility complex is signaled by GO as overrepresented in our cohort of cases.

There are two main limitations of the present study. First, the sample size is limited, although this work is the first of this kind in the field of RSV infection. We aimed at improving its power by selecting extreme phenotypes, assuming a larger effect of causal variants. Second, in lieu of an independent replication cohort we used alternative European control groups. The association findings could be consistently replicated in the different control groups, and the candidate genes observed in our study are also in line with previous evidence in the literature. Future genotyping and sequencing efforts would be necessary to obtain confirmatory evidence for the associations detected in the present study. It is also important to mention that allele frequencies for some of the candidate SNPs observed in the present study show differences between 1000G data and the known repository for exome sequencing data ExAC (http://exac.broadinstitute.org). For instance, for European non-Finish data set in ExAC, SNP rs199665292 has similar allele frequencies to our RSV patients; however, the exome data from Spanish controls in Dopazo *et al*.^42^ have allele frequencies that are close to IBS (Table S1).

Future studies based on larger samples could also address the genetic variation underlying sub-phenotypes of RSV infection (e.g. severity, length of hospital stay, asthma, etc)^74–76^. In addition, the strategy employed in the present study based on WES could be also of interest at investigating the presumed genetic link existing between RSV and asthma disease^75,77^.

## Conclusion

The present study is pioneering in analyzing exome sequence variation in RSV patients, and in identifying common and rare variants related to host susceptibility to RSV infection. Single-base association analyses suggest new candidate variants related to RSV infection. In addition, burden association tests indicate that rare variants with a high predicted pathogenicity are enriched in a number of genes in carriers when compared to controls. Some of the variants detected fall in regions that have previously been reported as candidate genes in RSV infection (HLA genes), while others, such as olfactory and taste receptor genes are newly reported here, and therefore open new horizons in the search for genetic factors related to RSV infection. To the best of our knowledge this is the first attempt at using WES in the context of the genetic predisposition to infectious disease in childhood by targeting severe phenotypes. This work demonstrates that WES can be particularly useful for the detection of signals of association related to rare variants, in contrast with traditional studies that focused on common disease variation.

## Electronic supplementary material

Supplementary Information

Supplementary Dataset
