# Supplementary material for: Whole Exome Sequencing reveals new candidate genes in host genomic susceptibility to Respiratory Syncytial Virus Disease
Source: Sci Rep. 2017 Nov 21;7:15888. doi: 10.1038/s41598-017-15752-4 (PMC5698448; doi:10.1038/s41598-017-15752-4)
Supplement: Supplementary file 1 — Supplementary Information [file 41598_2017_15752_MOESM1_ESM.pdf]

Supplementary Data

Whole Exome Sequencing reveals new candidate genes in host genomic susceptibility to Respiratory Syncytial Virus Disease

Antonio Salas, Jacobo Pardo-Seco, Miriam Cebey-López, Alberto Gómez-Carballa, Pablo Obando-Pacheco, Irene Rivero-Calle, María-José Currás-Tuala, Jorge Amigo, Jose Gómez-Rial, Federico Martinón-Torres, on behalf of GENDRES network (www.gendres.org)

Index

Figure S1. MDS analysis carried out on a matrix of pair-wise individual identity-by-state values between RSV patients *vs*. IBS data for the 1 to 8 dimensions.

Figure S2. Manhattan plot of RSV common SNP variants *versus* CEU, GBR, TSI and “ALL” controls groups.

Figure S3. Manhattan plot of IBS SNP variants *versus* CEU, GBR, and TSI. In red are those variants appearing as statistically significant between RSV and IBS controls.

Figure S4. QQ-plot of RSV common SNP variants in RSV *vs.* CEU, GBR, TSI and “ALL” controls groups

Figure S5. Linkage disequilibrium of *MUC4* variants.

Figure S1

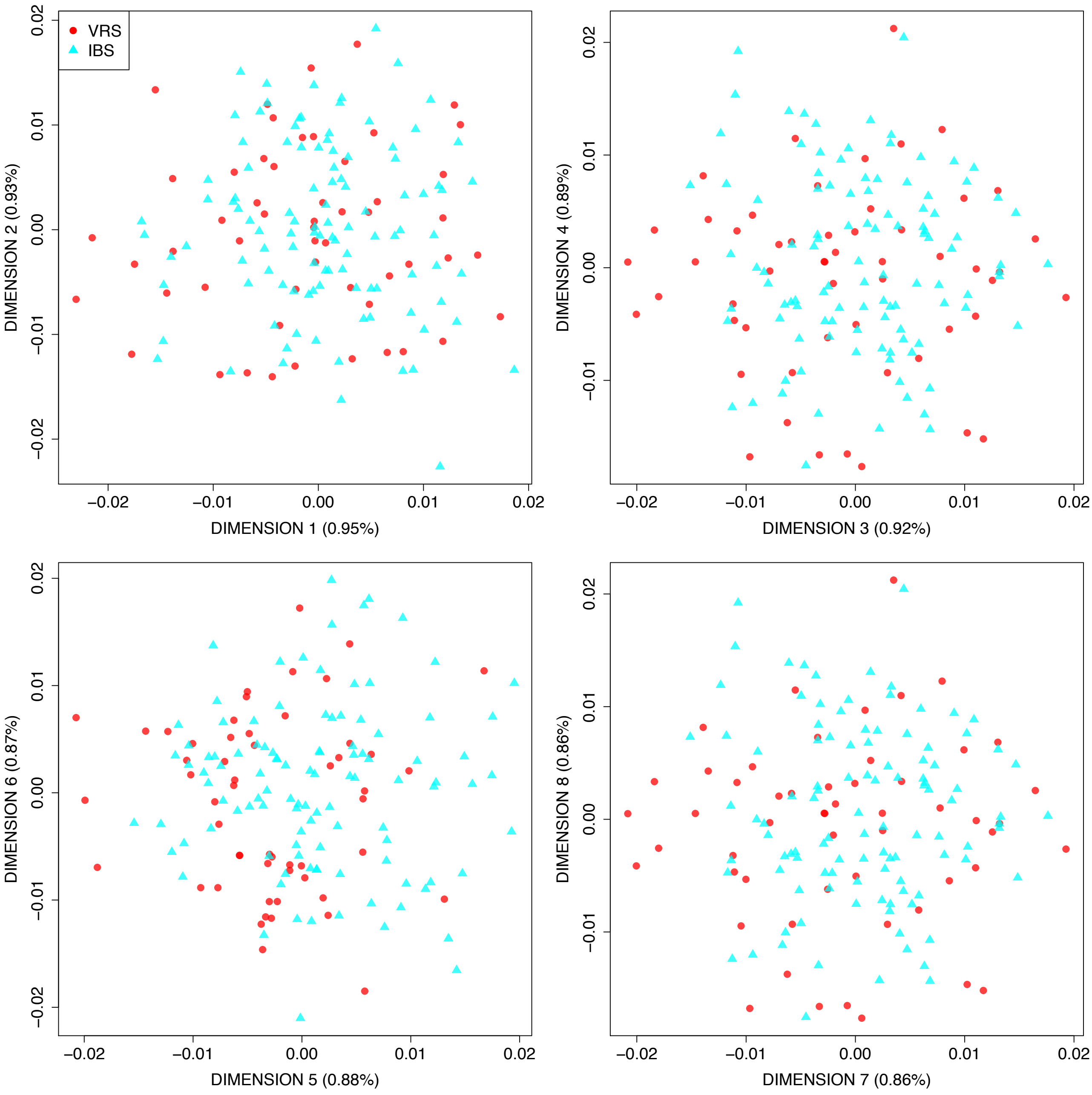
VRS
IBS
DIMENSION 1 (0.95%)
DIMENSION 2 (0.93%)
DIMENSION 3 (0.92%)
DIMENSION 4 (0.89%)
DIMENSION 5 (0.88%)
DIMENSION 6 (0.87%)
DIMENSION 7 (0.86%)
DIMENSION 8 (0.86%)
−0.02
−0.01
0.00
0.01
0.02

Figure S2

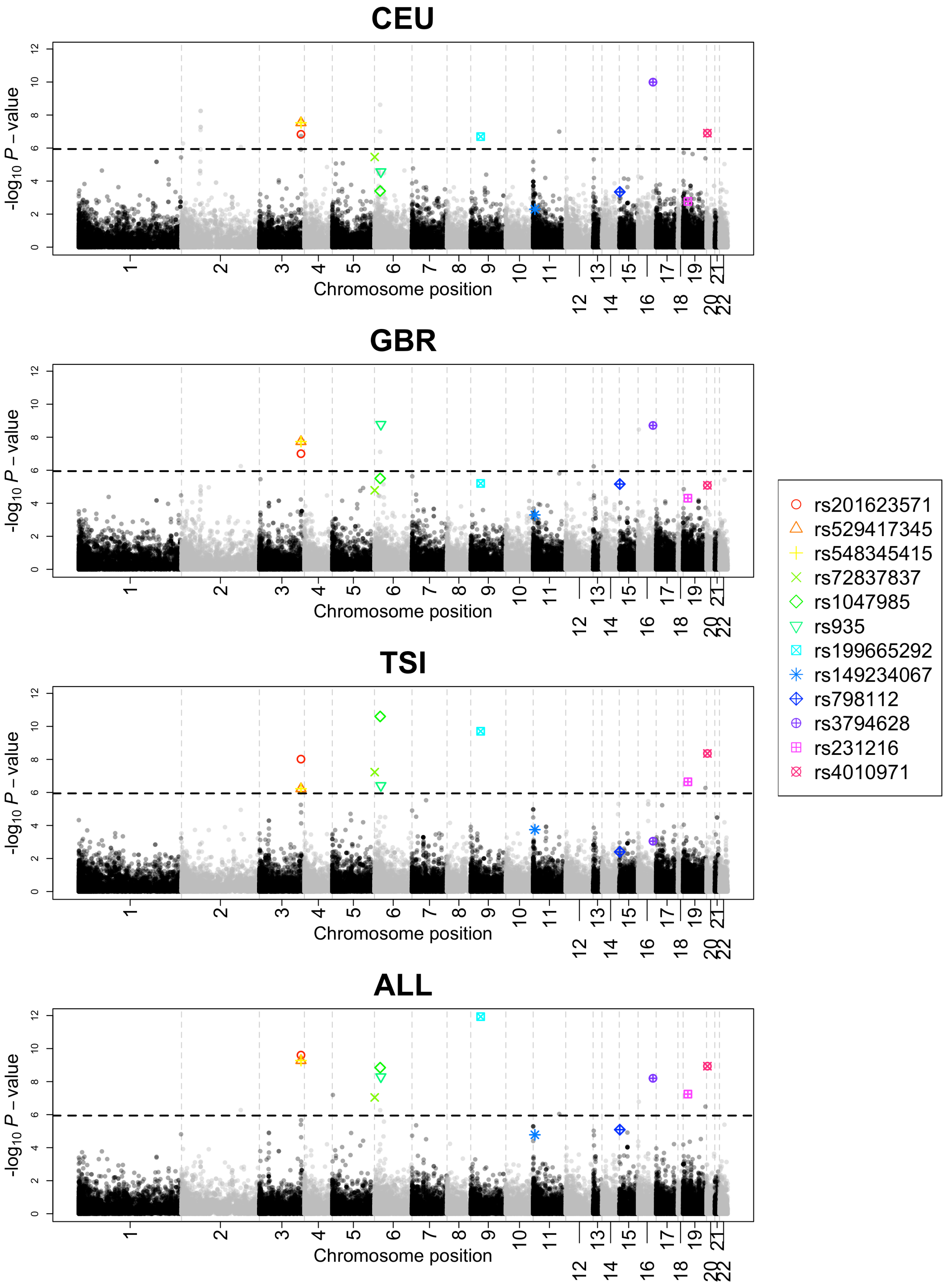
CEU
GBR
TSI
ALL
-log10 P – value
Chromosome position
1
2
3
4
5
6
7
8
9
10
11
12
13
14
15
16
17
18
19
20
21
22
rs201623571
rs529417345
rs548345415
rs72837837
rs1047985
rs935
rs199665292
rs149234067
rs798112
rs3794628
rs231216
rs4010971

Figure S3

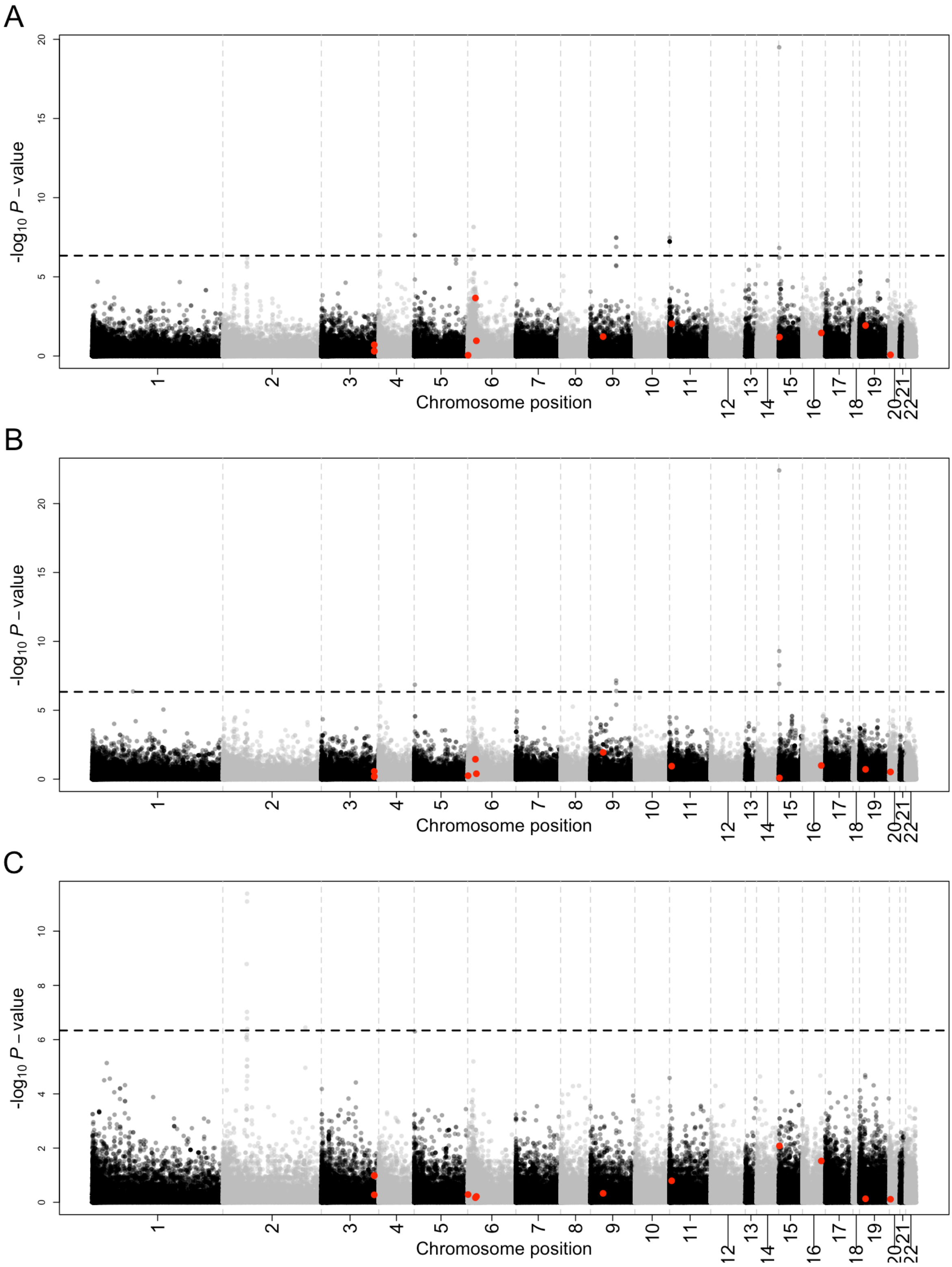
A
B
C
-log10 P – value
Chromosome position
1
2
3
4
5
6
7
8
9
10
11
12
13
14
15
16
17
18
19
20
21
22

Figure S4

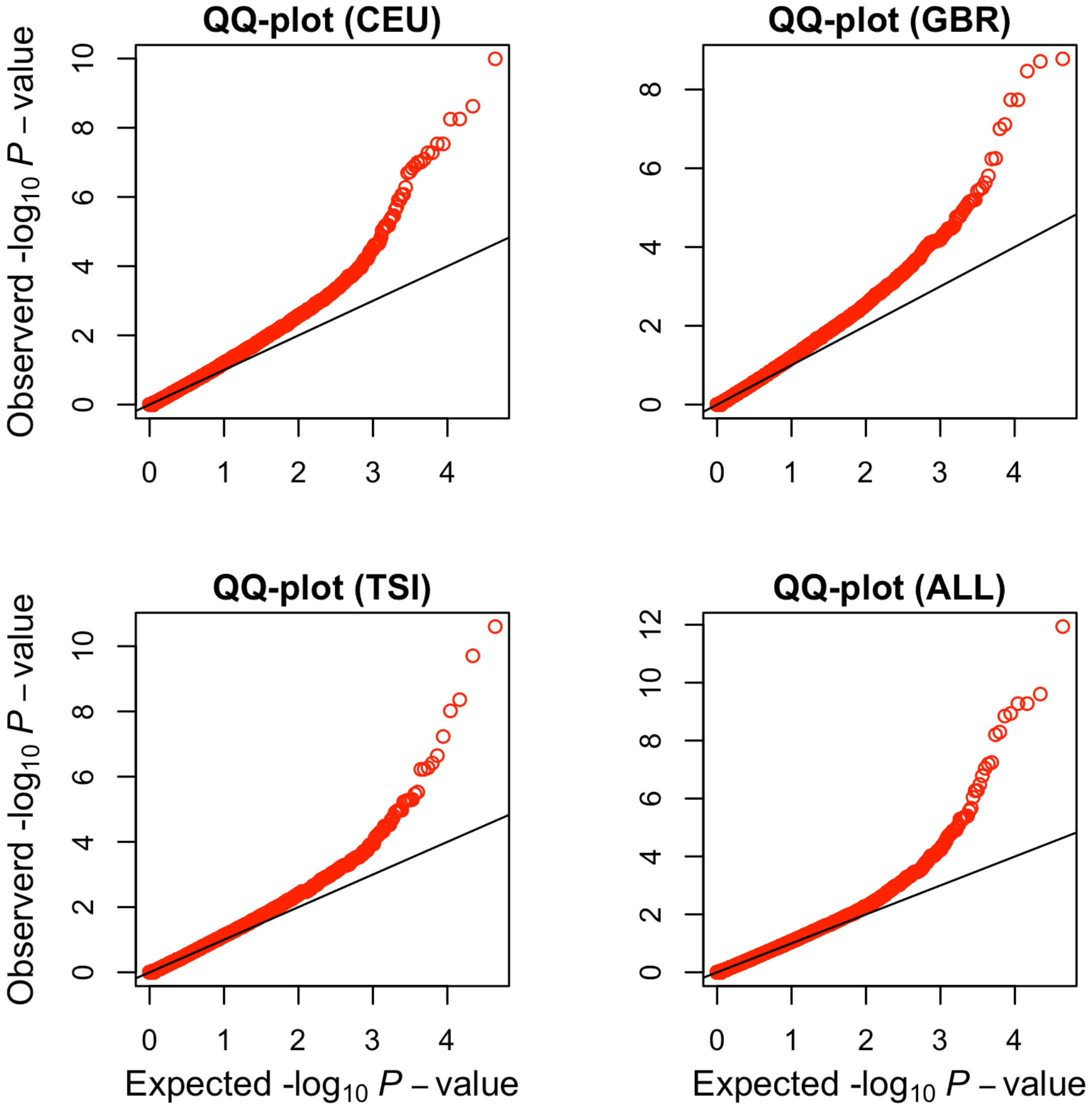

QQ-plot (CEU)
QQ-plot (GBR)
QQ-plot (TSI)
QQ-plot (ALL)
Observerd -log10 P – value
Expected -log10 P – value

Figure S5

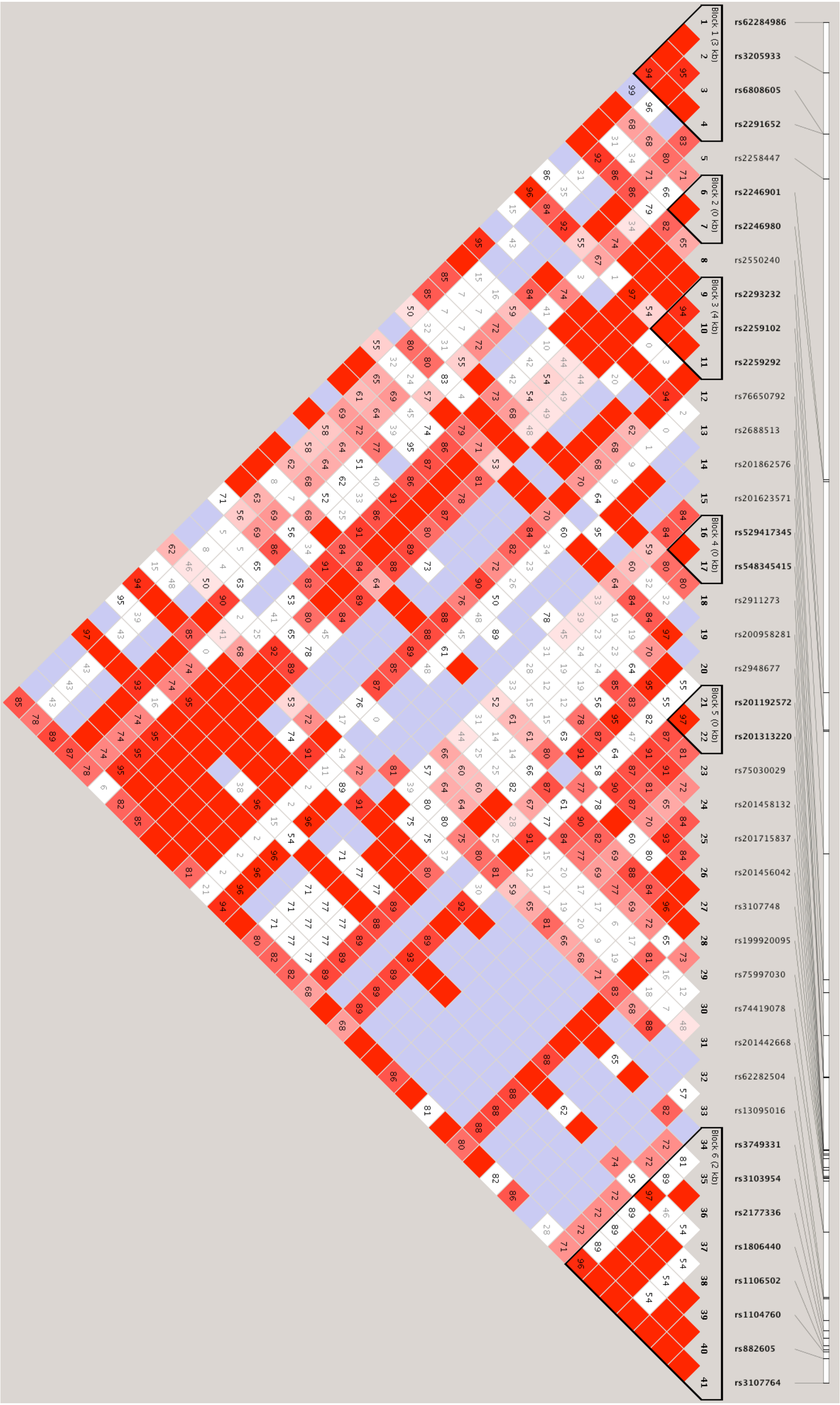
Block 1 (3 kb)
Block 2 (0 kb)
Block 3 (4 kb)
Block 4 (0 kb)
Block 5 (0 kb)
Block 6 (2 kb)
rs62284986
rs3205933
rs6808605
rs2291652
rs2258447
rs2246901
rs2246980
rs2550240
rs2293232
rs2259102
rs2259292
rs76650792
rs2688513
rs201862576
rs201623571
rs529417345
rs548345415
rs2911273
rs200958281
rs2948677
rs201192572
rs201313220
rs75030029
rs201458132
rs201715837
rs201456042
rs3107748
rs199920095
rs75997030
rs74419078
rs201442668
rs62282504
rs13095016
rs3749331
rs3103954
rs2177336
rs1806440
rs1106502
rs1104760
rs882605
rs3107764
